# Preparation and Properties of Chitosan Complexes Consisting of *Artemisia argyi* Volatile Oil Nanoemulsion

**DOI:** 10.3390/molecules30030585

**Published:** 2025-01-27

**Authors:** Shun Zhang, Kewei Zuo, Lijun Zhang, Chenlu Zhang, Juan Shi

**Affiliations:** 1 School of Biological Science and Engineering, Shaanxi University of Technology, Hanzhong 723000, China; 15929874564@163.com (S.Z.); keweizuo@163.com (K.Z.); 2 Shaanxi Province Key Laboratory of Bio-Resources, Hanzhong 723000, China; 3 Qinba State Key Laboratory of Biological Resources and Ecological Environment (Incubation), Hanzhong 723000, China; 4 School of Chemical and environmental Science, Shaanxi University of Technology, Hanzhong 723000, China; 18829368533@163.com; 5 Shaanxi Provincial Key Laboratory of Catalysis Basis and Application, Hanzhong 723000, China

**Keywords:** *Artemisia argyi* volatile oil, nanoemulsion, chitosan, antibacterial activity, in vitro release, antioxidant

## Abstract

*Artemisia argyi* volatile oil (AAVO) is a kind of natural oil with abundant active components and remarkable medicinal and healthcare value. However, AAVO has low solubility, stability, and bioavailability. Here, to address these issues, a nanoemulsion system of *Artemisia argyi* volatile oil (AAVO-Ne) is constructed using phase transition titration, and the conditions are continuously optimized to combine it with chitosan, forming a chitosan composite of the volatile oil nanoemulsion (AAVO-NeCs). The structure was analyzed using Fourier transform infrared (FT-IR) spectroscopy, and the performance was evaluated through in vitro antibacterial tests, in vitro release experiments, and antioxidant assays. The results indicated that the typical characteristic absorption peaks of AAVO shifted in the AAVO-Ne spectrum and new absorption peaks appeared in the AAVO-NeCs, which implied that the formation of AAVO-NeCs involved not only a physical encapsulation process but also certain chemical interactions, thus enhancing the stability and bioactivity of the composites. Compared to AAVO, AAVO-NeCs exhibited a 1.87-fold increase in antibacterial activity against antibiotic-resistant bacteria. Meanwhile, the in vitro release study demonstrated that AAVO-NeCs exhibited a biphasic release pattern. Compared to AAVO-Ne and AAVO, AAVO-NeCs also showed a significant enhancement in antioxidant activity. Overall, AAVO-NeCs demonstrate improved solubility and efficacy of AAVO, as well as high-efficiency delivery, antibacterial, sustained-release, and antioxidant properties. These attributes position AAVO-NeCs as a promising candidate for applications in drug delivery, food preservation, and other fields, offering innovative solutions and contributing to the sustainable development of related industries.

## 1. Introduction

In recent years, the misuse of antibiotics has led to an increasing issue of bacterial resistance, which has become a major global public health threat. Monitoring by the China Antimicrobial Resistance Surveillance Network in the first half of 2024 revealed that the emergence of resistant strains continues to rise, highlighting the urgent need for the discovery of new antimicrobial agents [1,2]. Essential oils from natural plants, due to their broad-spectrum antibacterial properties, show inhibitory effects against various bacteria, fungi, viruses, and parasites, and thus hold promise as effective antibacterial agents and antioxidants [3,4,5]. Studies by Sánchez et al. have demonstrated that essential oils from the Ricinus genus in Colombia exhibit significant antibacterial activity against methicillin-resistant *S aureus*, and ampicillin-resistant and streptomycin-resistant *E. coli* [6]. Furthermore, essential oils from Cassia bark, oregano, thyme and carnation also display notable antimicrobial activity against resistant *E. coli* [7,8,9]. These findings highlight the tremendous potential of plant essential oils in addressing bacterial resistance, offering new perspectives and methods for the development of novel antimicrobial agents.

*Artemisia argyi* H. Lév. & Vaniot, commonly known as Mugwort, is a plant species belonging to the Asteraceae family and the *Artemisia* genus [10,11,12,13,14]. *Artemisia argyi*, a traditional Chinese medicinal herb, exhibits significant pharmacological potential through its main bioactive component, *Artemisia argyi* volatile oil (AAVO) (Table 1). This oil primarily contains compounds such as eucalyptol, camphor, borneol, and caryophyllene, which contribute to its diverse pharmacological activities. These activities include antiviral, antibacterial, bronchodilatory, anti-inflammatory, analgesic, and sedative effects, demonstrating the wide therapeutic potential of AAVO in various medical applications [15]. Currently, the extraction methods for AAVO primarily include steam distillation, organic solvent extraction, supercritical carbon dioxide extraction, and microwave extraction. Common methods used for antimicrobial testing include the agar disk diffusion method, colony counting method, minimum inhibitory concentration (MIC) method, and minimum bactericidal concentration (MBC) method [16,17,18,19]. Current research indicates that the AAVO extracted via steam distillation demonstrates inhibitory effects against both Gram-positive and Gram-negative bacteria, as well as fungi, as shown by the agar disk diffusion assay. The proposed mechanism of action suggests that the AAVO disrupts the cell wall and membrane of the tested microorganisms, leading to the leakage of lipids, proteins, and other molecules, as well as the release of alkaline phosphatase, ultimately resulting in cell death. This highlights the natural antimicrobial potential of AAVO [20,21].

AAVO has low water solubility, a high evaporation rate, and unstable physical properties, which result in its poor bioavailability. In recent years, nanodrug delivery systems have been increasingly applied to encapsulate essential oils, aiming to improve their physicochemical properties. Commonly used nanodelivery systems for essential oils include nanoemulsions (Ne), microemulsions, microcapsules, and liposomes [22]. Ne, as an advanced essential oil delivery system, has garnered significant attention in the scientific literature. It offer advantages such as simple preparation, small particle size, large specific surface area, and kinetic stability, and can encapsulate and directly load active compounds. This makes it a highly attractive novel approach for incorporating plant-derived active extracts into food and pharmaceutical formulations [23,24,25]. In the study of volatile oil nanoemulsion, it was found that the MIC and MBC of the nanoemulsion were significantly lower compared to the pure volatile oil, indicating that the nanoemulsion system can enhance the antimicrobial activity of the volatile oil [26]. Studies have indicated that cinnamon essential oil nanoemulsion can be successfully synthesized using the water-in-oil method. By simulating the digestive process of the essential oil nanoemulsion in the gastrointestinal tract, the bioavailability of the essential oil in food and pharmaceuticals is enhanced. Both ABTS and DPPH radical scavenging assays, as well as the paper disc diffusion test, demonstrate that the nanoemulsion exhibits superior antioxidant and antimicrobial properties [27]. It can be concluded that by constructing an *Artemisia argyi* essential oil nanosuspension (AAVO-Ne), the solubility, bioavailability, and stability of AAVO can be significantly improved. This approach contributes to enhancing the therapeutic efficacy of AAVO and is an effective method to boost its performance [28,29,30].

Chitosan (Cs), a natural polysaccharide extracted from chitin, possesses various excellent physiological functions, such as biodegradability, biocompatibility, non-toxicity, and antimicrobial properties. Due to its wide range of applications, Cs has been successfully utilized in several fields, including food additives, textiles, cosmetics, and antimicrobial agents [31,32]. Studies have shown that encapsulating cinnamon essential oil into Cs nanolipid emulsions using ion gelation technology significantly enhances the oil’s efficacy, demonstrating excellent performance in terms of encapsulation efficiency, oil loading, in vitro release, bioactivity, and antioxidant properties [33]. The direct loading of essential oils into Cs can significantly enhance their antibacterial properties, indicating the method’s great potential for application in food preservation and antibacterial packaging [34,35]. However, research on the preparation of essential oils as nanoemulsions to alter their water solubility and bioavailability, and their subsequent complexation with Cs to enhance antibacterial properties, is relatively limited.

This study aims to prepare AAVO-Ne and incorporate Cs in order to significantly enhance their antibacterial, antioxidative, and controlled release properties. AAVO, as a natural active ingredient, exhibits various pharmacological activities, including antibacterial and antioxidative effects. However, its applications are limited due to issues such as poor stability and low water solubility. Ne technology can improve the stability and bioavailability of AAVO, while the addition of Cs is expected to further enhance its controlled release effect and antibacterial activity. By optimizing the preparation process of AAVO-Ne and combining the characteristics of Cs, this study aims to develop an efficient and stable AAVO-NeCs system. This approach not only provides a novel strategy for developing more effective natural antibacterial products, extending shelf life, and enhancing the efficacy of active ingredients in pharmaceuticals or cosmetics, but also facilitates the broader application of AAVO in hygiene, environmental, biomedical, and other fields.

## 2. Results and Discussions

### 2.1. Preparation and Quality Determination of AAVO-Ne

The phase inversion method in the preparation of nanoemulsions offers significant advantages, including low energy consumption, simple operation, cost-effectiveness, and strong environmental sustainability. Compared to high-energy methods, it does not require complex equipment and can prepare emulsions under mild conditions, making it suitable for the protection of volatile oil components [36]. During the preparation process, the role of surfactants is to cover a large interfacial area and reduce interfacial tension, thereby stabilizing the emulsion. If the concentration of surfactant is insufficient, it will not effectively lower the interfacial tension, leading to the breakdown of the emulsion structure and instability. However, when the surfactant concentration is too high, the intermolecular forces between emulsion droplets increase, promoting droplet collision and aggregation, ultimately leading to instability. Therefore, selecting an appropriate Km value is crucial for ensuring the stability of the emulsion and preventing droplet aggregation [37,38].

At different Km ratios, as shown in Table 2, the addition of varying amounts of AAVO results in the formation of different surfactant-to-cosurfactant ratios (SOR) in the AAVO-Ne system. Specifically, when the Km ratio is 4:1, AAVO-Ne forms effectively within the SOR range of 9:1 to 5:5. When the Km ratio is 3:1, AAVO-Ne forms within the SOR range of 9:1 to 6:4. At a Km ratio of 2:1, AAVO-Ne forms within the SOR range of 9:1 to 5:5. Furthermore, when Km is 1:1, AAVO-Ne can be obtained within the SOR range of 8:2 to 5:5. At Km = 1:2, AAVO-Ne formation is possible within the SOR range of 8:2 to 6:4. Similarly, when Km = 2:3, AAVO-Ne can also be successfully formed within the SOR range of 8:2 to 6:4.

**Figure 1:**
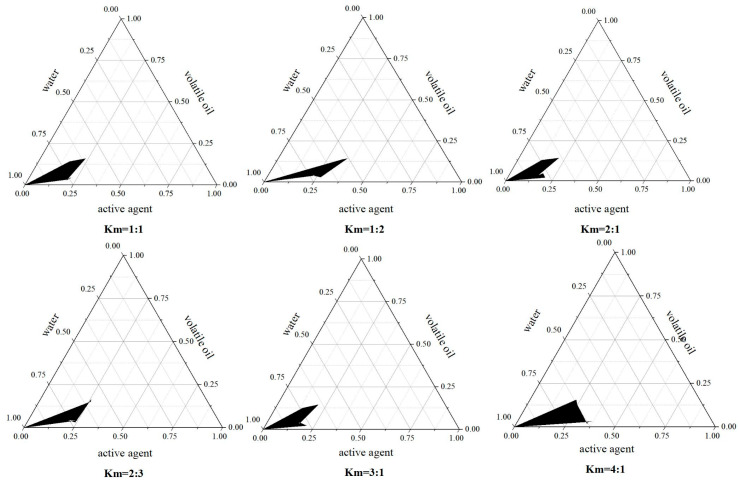
Quasi-ternary phase diagram of AAVO-Ne with different Km values (1:1, 1:2, 2:1, 2:3, 3:1, 4:1).

As shown in Figure 1, the ternary phase diagram analysis indicates that the emulsion region has the largest area when the Km ratio is 4:1. Compared to other Km values, at this ratio, the surfactant and co-surfactant can effectively form a stable micelle structure [39]. Based on this result, we chose Km = 4:1 as the optimal mass ratio of surfactant to co-surfactant for preparing the AAVO-Ne system.

As shown in Table 3, under the optimal Km = 4:1 conditions, AAVO-Ne can be successfully prepared with surfactant-to-co-surfactant ratios (SOR) of 9:1, 8:2, 7:3, 6:4, and 5:5. However, when the SOR is 8:2 and 7:3, the resulting AAVO-Ne does not meet the required specifications due to noticeable turbidity and low transparency. In contrast, when the SOR is 9:1, 6:4, and 5:5, the emulsions are clear and transparent. Among these, the AAVO content reaches its maximum at an SOR of 5:5. Therefore, a surfactant-to-co-surfactant ratio of 5:5 is selected to optimize the preparation process of AAVO-Ne.

Based on the above screening results, the optimal composition for preparing AAVO-Ne is as follows: 19.88% Tween-80, 3.11% anhydrous ethanol, 16.15% AAVO, 60.86% distilled water. The AAVO-Ne prepared with these mass percentages is transparent, clear, stable, and shows no phase separation.

The structural type of AAVO-Ne was determined through dyeing experiments, which revealed that methylene blue diffused faster in AAVO-Ne than Sudan III, indicating that the prepared AAVO-Ne is of the oil-in-water (O/W) type. Additionally, the pH of the prepared AAVO-Ne was measured using precision pH test paper, yielding a result of 5.5 ± 0.5, suggesting that the emulsion is mild and poses no safety risk to the skin. After one week of centrifugation, the AAVO-Ne showed no phase separation, emulsion breakdown, or crystallization, and there was no significant change in appearance. This indicates that the preparation process and stability of AAVO-Ne are suitable.

### 2.2. The Preparation of AAVO-NeCs

**Figure 2:**
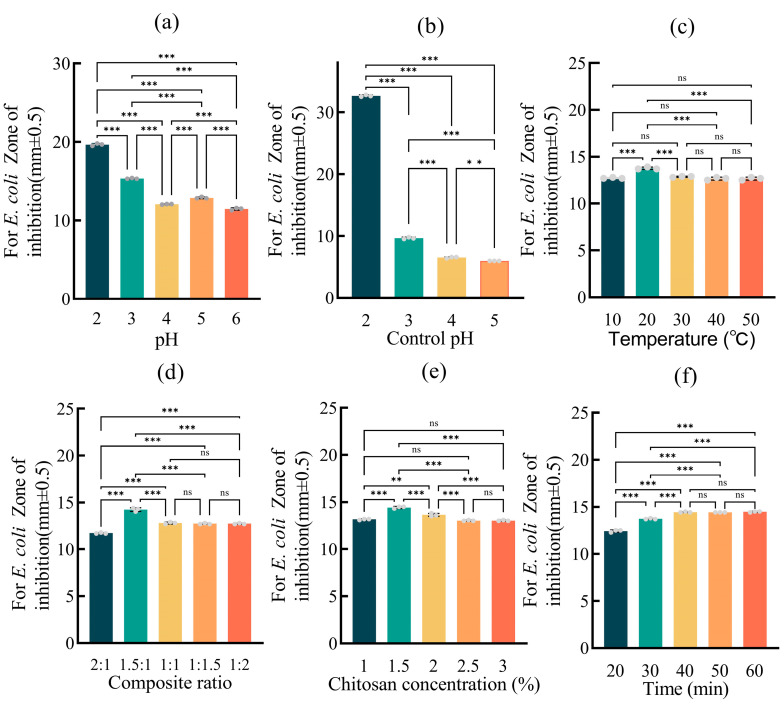
Analysis plot of AAVO-NeCs prepared from the one-factor experiment. (**a**) pH variation; (**b**) pH control (pure pH); (**c**) Temperature variation; (**d**) Variation of the complex ratio; (**e**) Variation of chitosan concentration; (**f**) Variation of complexation time. (**, *p* < 0.01; ***, *p* < 0.001, ns, no significance).

The preparation conditions of the AAVO-NeCs were optimized through a single-factor experiment, and the antibacterial activity of the AAVO-NeCs against *E. coli* was evaluated using the paper disc diffusion method. The antibacterial effect under different conditions was studied with the diameter of the inhibition zone as the indicator in Figure 2 (Supplementary Figure S2). In the pH adjustment experiment, the results of single-factor significance analysis indicated that the diameter of the inhibition zone was not linearly related to the pH value. Specifically, the AAVO-NeCs showed the best antibacterial effect at pH 2, with the inhibition zone diameter close to 20 mm. As the pH increased, the inhibition zone gradually shrank, although there was a slight recovery at pH 5 before it decreased again (Figure 2a). Comparing the inhibition effect of pure acetic acid solution on bacterial growth at different pH values, it was found that the inhibition zone diameter significantly increased when the pH was adjusted from 3 to 2, while at pH 4 and 5, the acid’s inhibitory effect on bacterial growth was milder (Figure 2b). Some studies have also shown that under conditions of pH 2.0, *E coli* requires the presence of glutamic acid, arginine, or lysine to survive, as these amino acids help neutralize the acidic intracellular environment through their decarboxylation [40]. Therefore, to exclude the impact of acidity on bacterial growth and to ensure the AAVO-NeCs is non-irritating to the skin, pH 5 was selected as the optimal condition.

The effect of temperature on the antibacterial activity of the AAVO-NeCs was also studied. The results showed that the AAVO-NeCs prepared at 20 °C had significantly higher antibacterial activity than those prepared at other temperatures. At 10 °C, 30 °C, 40 °C, and 50 °C, the antibacterial activity of the composite showed no significant differences (Figure 2c). This suggests that the antibacterial activity of the AAVO-NeCs is relatively stable across different temperatures, with the best effect observed at 20 °C.

The effect of the ratio of the Cs acetic acid solution on AAVO-Ne and the Cs concentration on the antibacterial activity of the AAVO-NeCs was also investigated. It was found that as the ratio of AAVO-Ne and the Cs concentration increased, the antibacterial activity of the composite first increased and then decreased, stabilizing within a certain concentration range (Figure 2d,e). This result indicates that there is an optimal ratio between the AAVO-Ne and Cs concentrations and increasing the ratio does not necessarily improve the activity. Further analysis revealed that the composite exhibited the best antibacterial performance when the ratio of AAVO-Ne to Cs acetic acid solution was 1.5:1 and the Cs concentration was 1.5%.

Finally, the effect of ultrasound treatment time on the antibacterial activity of the AAVO-NeCs was studied. The results showed that within a certain time range, the antibacterial activity of the AAVO-NeCs increased with the extension of ultrasound treatment time and stabilized after 40 min. Significance analysis indicated that after 40 min, the antibacterial effect of the AAVO-NeCs was no longer significantly affected by further extension of the ultrasound time (Figure 2f). Therefore, 40 min was chosen as the optimal ultrasound treatment time.

In conclusion, the optimal preparation conditions for the composite of AAVO-Ne and Cs acetic acid solution are as follows: AAVO-Ne and Cs acetic acid solution mixed at a 1.5:1 ratio, pH adjusted to 5, temperature set at 20 °C, and ultrasound treatment for 40 min. Under these conditions, the AAVO-NeCs demonstrated the best antibacterial activity.

When the pH was adjusted to an alkaline level, the material was found to coagulate into flocculent precipitates due to structural changes induced by the alkaline environment. This phenomenon can be attributed to the sensitivity of Cs solubility to pH. Under acidic conditions, the amino groups of Cs become protonated, thereby increasing its solubility, which facilitates its interaction with AAVO-Ne [41,42]. Therefore, a pH range of 2 to 6 was chosen for the study. Considering the effects of acidic substances on bacterial growth, and in order to ensure both the antibacterial efficacy and gentle, non-irritating properties of AAVO-NeCs on the skin, a final pH of 5 was selected for preparation.

Temperature also significantly influences the stability of AAVO-NeCs. Previous studies have shown that an increase in temperature leads to the degradation of Cs, while the components of AAVO may volatilize, thereby increasing the instability of AAVO-NeCs [43]. On the other hand, at very low temperatures, Cs may gel, causing AAVO molecules to aggregate, thus reducing the uniformity and stability of the emulsion. Furthermore, studies indicate that an increase in temperature can reduce the viscosity of Cs, likely due to the activation of molecules within a certain temperature range, which promotes effective collisions between molecules and the cleavage of glycosidic bonds. Therefore, AAVO-NeCs were prepared at 20 °C to maintain their stability and effectiveness, achieving optimal antibacterial performance.

The content of AAVO-Ne plays a crucial role in the properties of AAVO-NeCs, with different preparation results depending on the ratio. Research shows that too high a content of AAVO-Ne can lead to increased volatility, affecting the release rate and duration of the nanoemulsion, while too low a content may be insufficient for achieving the desired active effect. AAVO-Ne and Cs function complementarily. For instance, when Cs and pectin are used to prepare nanoemulsions for localized delivery of ethylhexyl triazine, it was found that low concentrations of Cs combined with varying percentages of pectin can enhance the stability of the nanoemulsion. This indicates that the stability of the nanoemulsion is closely related to the ratio of Cs and AAVO-Ne, and excessive use of one component can interfere with the function of the other, thereby reducing the overall effectiveness [44].

The concentration of Cs also has a significant impact on the viscosity and dispersion of AAVO-NeCs. High concentrations of Cs can increase the viscosity of the solution, affecting the uniform dispersion of AAVO-Ne and potentially leading to the formation of uneven AAVO-NeCs. Conversely, low concentrations of Cs may fail to form a sufficient network structure, resulting in insufficient strength of AAVO-NeCs, thereby affecting their stability [45,46].

Moreover, ultrasonic treatment has an important effect on the stability and shelf life of the nanoemulsion. Studies have shown that the ultrasonic treatment time significantly affects the particle size, turbidity, and dispersion of soybean protein isolate–phosphatidylcholine nanoemulsions. Extending the ultrasonic treatment time appropriately can improve the dispersion and stability of the emulsion [47]. However, excessive ultrasonic treatment time may lead to an increase in particle size, thereby altering the physical properties of AAVO-NeCs, such as viscosity and rheology, ultimately affecting the performance of the material. Considering both timeliness and performance, 40 min was selected as the optimal preparation time.

In conclusion, the preparation of AAVO-NeCs is influenced by factors such as the ratio of AAVO-Ne to Cs, ultrasonic treatment time, and Cs concentration. These factors interact with each other and collectively determine the stability and bioactivity of AAVO-NeCs.

### 2.3. FT-IR Analysis

**Figure 3:**
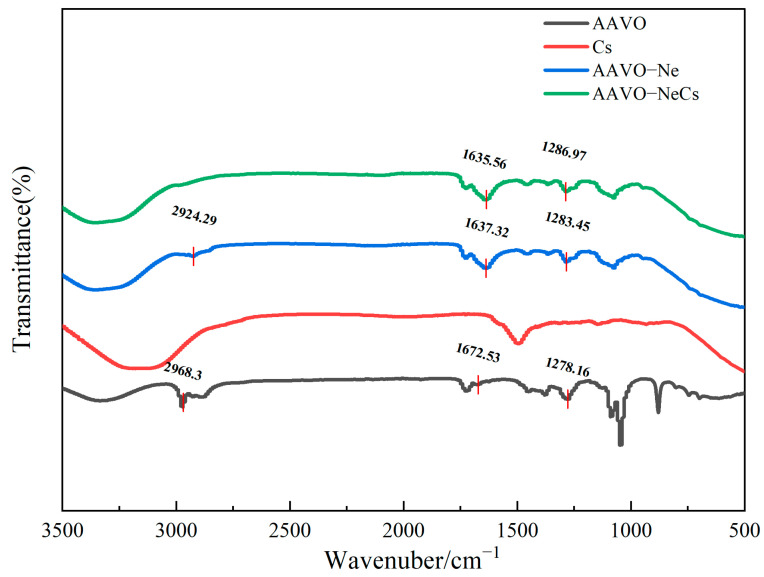
Infrared spectra of AAVO, AAVO-Ne and AAVO-NeCs.

FT-IR was used to test the structures of AAVO, AAVO-Ne, and AAVO-NeCs. The spectral analysis revealed distinct structural features in the characteristic absorption peaks of AAVO and AAVO-Ne. In the FT-IR spectrum of AAVO, as shown in Figure 3, the –OH stretching vibration peak around 3350 cm^−1^, the –CH_2_ stretching vibration peak near 2900 cm^−1^, and the C=O absorption peak at 1670 cm^−1^ [48], indicate the presence of its main active ingredients and functional groups. Compared to AAVO, the FT-IR spectrum of AAVO-Ne shows slight shifts in the peak positions, suggesting that interactions between the AAVO and the surfactant lead to minor changes in the chemical structure. In the AAVO-NeCs spectrum, new absorption peaks appear, indicating that hydrogen bonding or other interactions might occur between the amino groups of Cs and components in AAVO. These changes suggest that the formation of AAVO-NeCs involves not only physical encapsulation but also possible chemical interactions, which enhance the stability and biological activity of the complex. In summary, the FT-IR analysis results show that there are certain structural changes in AAVO, AAVO-Ne, and AAVO-NeCs, and the formation of AAVO-NeCs significantly affects the molecular structure of the complex, contributing to its improved biological activity and drug-release properties.

### 2.4. Antibacterial Detection of AAVO, AAVO-Ne and AAVO-NeCs

**Figure 4:**
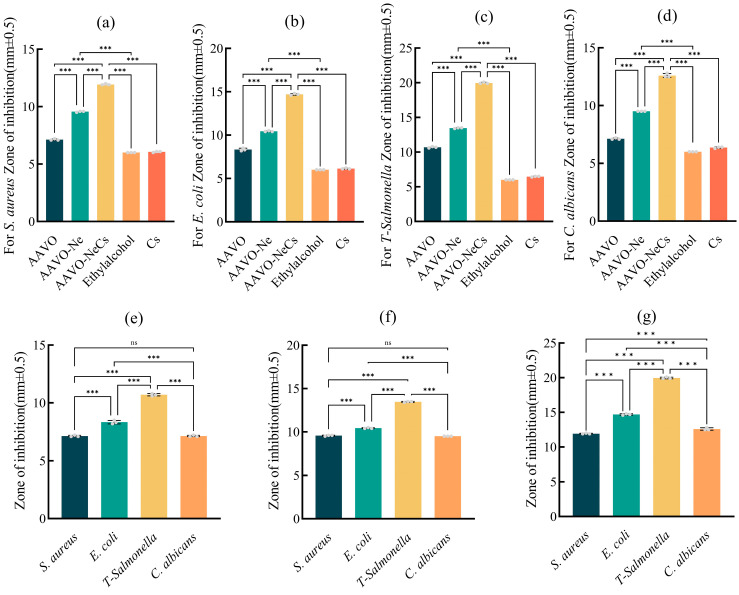
(**a**–**d**) Comparison analysis of the inhibitory effects of AAVO, AAVO-Ne, and AAVO-NeCs on the same type of bacteria. (**e**–**g**) Comparison analysis of the inhibitory effects of AAVO, AAVO-Ne, and AAVO-NeCs on different types of bacteria (***, *p* < 0.001, ns, no significance).

Under the same concentration conditions, the antibacterial effects of AAVO, AAVO-Ne, and AAVO-NeCs on *S. aureus*, *E. coli*, *T-Salmonella* and *C. albicans* were tested using the paper disc diffusion method, with 1.5% Cs acetate solution and absolute ethanol as controls. The results are shown in Figure 4, indicating that all three materials had an inhibitory effect on the growth of the four bacterial strains, with varying degrees of inhibition depending on the bacterial species. Horizontal analysis reveals that, except for the control group, the inhibitory effects of the three materials on the four bacteria increase in sequence (Figure 4a–d). Compared to AAVO, the inhibitory effect of AAVO-NeCs onS *T-Salmonella* increased from 10.7 mm to 20 mm, representing a 1.87-fold improvement (Figure 4c). Vertical analysis shows that AAVO-NeCs significantly enhanced the antibacterial activity against all four bacteria compared to AAVO. The effects of AAVO and AAVO-Ne on *S. aureus* and *C. albicansus* were not significantly different, but AAVO-NeCs demonstrated an improved inhibitory effect on *T-Salmonella* (Figure 4e,f). This enhanced effect is primarily attributed to the natural antibacterial properties of Cs, which boost the antibacterial activity of AAVO. Additionally, the volatile compounds in AAVO are capable of effectively penetrating bacterial cell walls and interfering with metabolic processes, thus allowing AAVO-NeCs to overcome the resistance seen with conventional antimicrobial agents and exhibit stronger inhibition. In summary, AAVO-NeCs demonstrated a significant advantage in antibacterial activity, particularly against *T-Salmonella*, showing a remarkable enhancement compared to AAVO alone. This suggests that AAVO-NeCs hold potential for application in antimicrobial fields, especially in combating antibiotic-resistant bacteria (Supplementary Figure S3) [49,50].

Through preliminary experiments and adjustments, it was observed that as the volume of the three materials increased, the turbidity of the suspension in the test tube gradually decreased until the solution became transparent. This indicates that all three materials exhibit antibacterial activity against *E. coli*, with the MIC values being 20.995, 17.765, and 4.307 μg/mL, respectively. The MIC of AAVO-NeCs was 4.8 times higher than that of AAVO, and the MIC of AAVO-NeCs was significantly higher compared to both AAVO and AAVO-Ne (Supplementary Figure S4).

**Figure 5:**
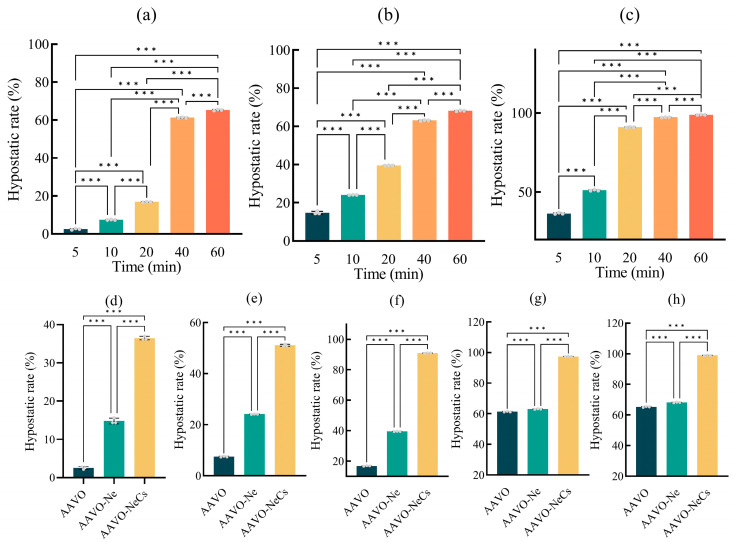
(**a**–**c**) The comparative analysis of the inhibition rates of AAVO, AAVO-Ne, and AAVO-NeCs against *E coli* at different time intervals. (**d**–**h**) The comparative analysis of the inhibition rates of AAVO, AAVO-Ne, and AAVO-NeCs against *E coli* at 5, 10, 20, 40, and 60 min, respectively. (***, *p* < 0.001).

To further investigate the antibacterial effects of the three materials on the test bacteria, colony counting was used to quantitatively assess the antibacterial activity over different time periods. As the exposure time of the bacteria to the materials increased, the number of colonies on the solid medium gradually decreased compared to the blank control group, eventually nearly disappearing. The calculation of the antibacterial activity of three materials against *E coli* at different time intervals showed significant differences in their effectiveness at each time point. The results are shown in Figure 5, the antibacterial rates of AAVO at 5, 10, 20, 40, and 60 min were 2.51%, 7.45%, 16.84%, 61.26%, and 65.33%, respectively. In comparison, AAVO-Ne exhibited higher antibacterial rates at the same time points, with values of 14.82%, 24.11%, 39.52%, 63.09%, and 68.20%. In contrast, AAVO-NeCs showed even more significant antibacterial effects, with antibacterial rates of 36.49%, 51.11%, 91.06%, 97.33%, and 98.94%. These results indicate that AAVO-NeCs outperformed the other two materials in terms of antibacterial performance, and its antibacterial effect gradually increased over time (Supplementary Figure S5).

A horizontal analysis of the antibacterial effects of the three materials at different exposure times shows that, as the exposure time increases, the inhibition rate against *E coli* gradually increases (Figure 5a–c). However, the difference in their inhibitory effects is not particularly large. With the increase in exposure time, especially at 20 min, AAVO-NeCs achieved an inhibition rate of over 90%, while AAVO and AAVO-Ne did not reach this level even after 1 h. Compared to AAVO, the antibacterial rate at the same time point was 5.5 times higher, and compared to AAVO-Ne, it was 2.3 times higher. A vertical analysis of the antibacterial effects at the same time points shows that AAVO-NeCs consistently exhibited significantly higher antibacterial activity than both AAVO and AAVO-Ne (Figure 5d–h). This result is consistent with the findings from the paper disc diffusion test and MIC experiments, further confirming that AAVO-NeCs possess excellent antibacterial performance.

The results of the removal of *E. coli* biofilm on cucumber slices indicated that all three materials were able to remove the biofilm to varying degrees (Supplementary Figure S6). Among them, AAVO-NeCs exhibited the strongest ability to remove *E. coli* biofilm on the cucumber surface, and it is naturally safe, making it highly promising for use in food preservation and shelf-life extension.

In summary, the antibacterial activity of the three materials can be ranked as follows: AAVO-NeCs > AAVO-Ne > AAVO, further proving that AAVO-NeCs possesses excellent biological activity.

AAVO contains various bioactive compounds, such as phenols, aldehydes, alcohols, and ketones, which give AAVO its natural antibacterial, antifungal, and antiviral properties. These compounds can disrupt bacterial cell membranes, inhibit enzyme activity, and interfere with microbial metabolic processes. AAVO-Ne enhances the antimicrobial effect by increasing the specific surface area and improving bioavailability, allowing AAVO to more effectively penetrate bacteria and interact with them. At the same time, Cs protects AAVO-Ne from degradation by light, heat, and oxygen, prolonging its stability, controlling the release rate, improving its bioavailability, and enhancing antimicrobial activity. The progressive increase in antibacterial activity across the three materials can be attributed to the synergistic effect between AAVO-Ne and the AAVO-NeCs [51,52,53]. Previous studies have shown that the combination of AAVO-Ne and Cs can create a synergistic effect, enhancing the antimicrobial performance of the complex substance through the interaction of active ingredients [54]. The preparation of AAVO-Ne enhances the dispersibility of AAVO, allowing it to be evenly distributed within the system, which significantly improves its solubility. Additionally, Cs as a natural polysaccharide, possesses excellent biocompatibility and sustained-release properties. Cs can form a protective film that encapsulates the AAVO in AAVO-Ne, preventing its rapid degradation and inactivation in the environment, thus prolonging the drug’s duration of action. Moreover, Cs has certain adhesive properties, which can enhance the drug’s retention time on mucosal surfaces and further improve its absorption efficiency. These factors work synergistically to significantly increase the bioavailability of AAVO-NeCs. Therefore, the increasing antibacterial effect of AAVO in AAVO-Ne and AAVO-NeCs reflects the interaction between the surface characteristics of the materials and the active components. This further demonstrates that AAVO-NeCs effectively enhances the stability and bioavailability of AAVO, thereby significantly improving its overall antibacterial performance.

### 2.5. AAVO, AAVO-Ne and AAVO-NeCs In Vitro Release Assay

**Figure 6:**
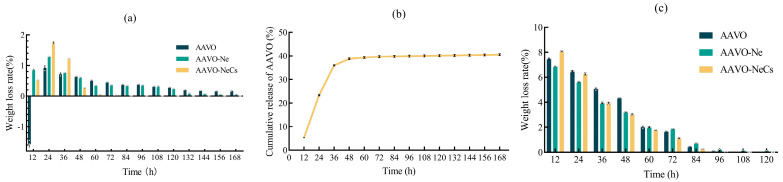
(**a**) The weight loss rate of AAVO, AAVO-Ne, and AAVO-NeCs at different time intervals; (**b**) in vitro release profile of AAVO-NeCs; (**c**) comparison of the weight loss rate of AAVO, AAVO-Ne, and AAVO-NeCs on freshly cut potato blocks.

In the in vitro release experiment, as shown in Figure 6a the initial volatilization loss rates of the three materials exhibited abnormal behavior, especially within the first 12 h, where the loss rate of some materials even became negative. This phenomenon is likely due to the strong water absorption ability of AAVO, which may have absorbed moisture released during the volatilization process of AAVO-Ne and AAVO-NeCs, resulting in an increase in its own weight. After 24 h, the experimental behavior stabilized, and the volatilization loss rates of AAVO, AAVO-Ne, and AAVO-NeCs gradually decreased, showing the expected release trend. After 48 h, AAVO-NeCs exhibited a particularly significant release effect, with its loss rate being noticeably lower than that of AAVO and AAVO-Ne. By 60 h, the loss rate of AAVO-NeCs approached zero, indicating that this material has good in vitro sustained release performance.

Further analysis of the cumulative release rate of AAVO-NeCs reveals that it initially undergoes a rapid release phase, followed by a controlled-release phase, as shown in Figure 6b. This biphasic release pattern is consistent with the results of Singh et al. in their study on the in vitro release of Cs and cinnamon essential oil-based novel nanoemulsions [33]. The membrane structure formed by AAVO-NeCs effectively limits the rapid release of essential oils, and the formation of the membrane slows the diffusion rate of the essential oil molecules, resulting in a more stable release process. The combination of the hydrophilic and hydrophobic regions of Cs enables it to respond to changes in different environmental conditions (such as pH, temperature, etc.), thus achieving on-demand release. This mechanism gives AAVO-NeCs excellent sustained-release and controlled-release properties, demonstrating its potential for widespread applications in drug delivery, food preservation, and cosmetics [55,56].

### 2.6. Antioxidant Detection of AAVO, AAVO-Ne and AAVO-NeCs

After the harvest of potato cubes, the weight loss rate increases with the extension of storage time, primarily due to water loss. After harvesting, fruits and vegetables gradually lose mass due to water evaporation, and this water loss process may also lead to physiological metabolic disturbances, affecting their freshness. The results in Figure 6c showed that the three materials protected freshly cut potato pieces compared with the control group. In particular, after 36 h of storage, the protective effect of AAVO-NeCs becomes more pronounced. AAVO-NeCs can effectively prevent the antioxidant components in AAVO from being oxidized by forming a membrane structure, thus improving its stability. This is because AAVO is rich in natural antioxidant compounds, such as phenolic compounds, flavonoids, and terpenes, which can effectively scavenge free radicals and inhibit oxidation reactions. Additionally, Cs itself has certain bioactive and antioxidant properties, with active functional groups like amino and hydroxyl groups that can react with free radicals, slowing down the oxidation process. The combination of these two components creates a synergistic effect, significantly enhancing the overall antioxidant capacity [57,58].

## 3. Materials and Methods

### 3.1. Materials

Anhydrous ethanol (AR, Lianlonghua Pharmaceutical Chemical Co., Ltd., Tianjin, China); Tween-80 (CP, Tianjin Fuyu Fine Chemical Co., Ltd., Tianjin, China); glacial acetic acid, sodium chloride (AR, Tianjin Tianli Chemical Reagents Co., Ltd., Tianjin, China); tryptone, agar, yeast extract powder (BR, Tianjin Tianli Chemical Reagents Co., Ltd., Tianjin, China); Chitosan (McLean Biochemical Technology Co., Ltd., Shanghai, China); *E. coli*, *S. aureus*, *C. albicans*, and *T-Salmonella* (Edible Fungi Research Institute, Shaanxi, China).

### 3.2. Preparation of AAVO

The *Artemisia argyi* leaves were sourced from the local market in Hanzhong City and identified by Professor Chenlu Zhang from the School of Biological Science and Engineering and Professor Juan Shi from the School of Chemistry and Environmental Science at Shaanxi University of Technology. The sample processing involved cleaning, trimming, drying at 35 °C for 30 min, pulverizing, and sieving through a 60-mesh screen. Several commonly reported extraction methods were evaluated, and one extraction method was selected for subsequent experiments based on yield calculation (Supplementary Figure S1) [59,60,61].

### 3.3. Preparation and Quality Assessment of AAVO-Ne

The AAVO-Ne system is constructed using phase transfer titration, a method that effectively controls the phase interface, enhancing the uniformity and stability of the nanoemulsion. This approach also improves its biocompatibility and drug-release properties. It offers high reaction efficiency with low energy consumption, making it more environmentally friendly. Additionally, it is applicable for the preparation of nanoemulsions with various active ingredients, demonstrating good adaptability [30].

In this study, Tween-80 was used as the surfactant and anhydrous ethanol as the co-surfactant, with various mass ratios (Km) (4:1, 3:1, 2:1, 1:1, 1:2, 2:3) prepared by thorough mixing. The mass ratio of AAVO to the surfactant–co-surfactant combination (SOR) was subsequently varied, with ratios of 9:1, 8:2, 7:3, 4:6, and 5:5, and the mixtures were thoroughly homogenized. Under the conditions of 20 °C and 650 rpm magnetic stirring, distilled water was added dropwise. The point of phase transition was determined when the emulsion transitioned from a clear to turbid appearance and then back to clear [62].

The quality of the emulsion was assessed based on transparency, clarity, and appropriate viscosity. A pseudo-ternary phase diagram was constructed using the mass fractions of the three components to determine the optimal Km value. Based on the optimal Km value, the effect of different mass ratios of the surfactant–co-surfactant combination and AAVO on the emulsion quality was evaluated to select the best formulation. The criteria for emulsion quality were clarity, transparency, appropriate viscosity, and no phase separation [63,64].

The structural type of the emulsion was identified using Sudan III and methylene blue staining methods. Specifically, 2 g of Sudan III and methylene blue were added to the emulsion, and the diffusion rates of both dyes were observed. A faster diffusion rate of Sudan III indicated that the emulsion was of the water-in-oil (W/O) type, while a faster diffusion rate of methylene blue suggested that the emulsion was of the oil-in-water (O/W) type. The pH of the prepared AAVO-Ne emulsion was measured using precision pH paper. One week later, we observed its appearance by centrifugation to assess its stability, with three parallel measurements conducted to ensure accuracy [65].

### 3.4. Preparation of AAVO-NeCs

Preparation conditions for the composite: A 1% *v*/*v* acetic acid aqueous solution was combined with varying amounts of Cs to obtain Cs solutions at different concentrations (1%, 1.5%, 2%, 2.5%, 3%). The mixture was stirred at 800 rpm using magnetic stirring until a transparent and homogeneous solution was achieved. The Cs solutions were then mixed with the AAVO-Ne at different mass ratios (2:1, 1.5:1, 1:1, 1:1.5, 1:2). The resulting mixtures were subjected to ultrasonic treatment for varying durations (20, 30, 40, 50, 60 min) at different temperatures (10, 20, 30, 40, 50 °C) and pH values (2, 3, 4, 5, 6). The optimal preparation conditions were determined based on the inhibitory activity against *E coli* using the paper disk diffusion assay.

### 3.5. Fourier Transform Infrared Spectroscopy Analysis

AAVO, AAVO-Ne and AAVO-NeCs were characterized by Fourier transform infrared spectroscopy. (Model: VERTEX 70; Manufacturer: Bruker, Ettlingen, Germany; Main Test Range:187,500 px^−1^~9250 px^−1^; Resolution: 4 px^−1^).

### 3.6. Antibacterial Experiment Research

#### 3.6.1. Activation and Culture of Bacteria

Activation of microbial strains: To prepare the growth medium, 8 g of tryptone, 5 g of yeast extract, 10 g of sodium chloride, and 30 g of agar were dissolved in 1 L of deionized water. The mixture was sterilized in an autoclave at 121 °C for 30 min. Under sterile conditions, the hot medium was poured into pre-sterilized petri dishes and allowed to cool and solidify for later use. A sterile inoculation loop was used to transfer a small amount of bacterial stock solution onto the surface of Luria-Bertani (LB) agar plates, which were then incubated at 37 °C in a light-controlled incubator for 12 h. After incubation, individual bacterial colonies were selected and transferred into test tubes containing 5 mL of liquid medium. These tubes were then incubated at 37 °C with constant shaking for 12 h before being stored in a refrigerator for future use.

Bacterial cultivation: *E. coli*, *T-Salmonella*, *S. aureus* and *C. albicans* were cultured in LB medium at 37 °C. The bacterial suspensions were adjusted to a concentration of 10^5^ CFU (colony-forming units)/mL for subsequent antibacterial testing [66].

#### 3.6.2. Antibacterial Experiment

Paper disc diffusion method experiment: In this experiment, *S. aureus*, *E. coli*, *T-Salmonella* and *C. albicans* were selected as test bacteria. The paper disc diffusion method was used to qualitatively evaluate the antibacterial properties of AAVO, AAVO-Ne, and AAVO-NeCs. All experimental materials were autoclaved at 121 °C for 20 min. AAVO, AAVO-Ne, and AAVO-NeCs were dissolved in 170 μg/mL solutions using absolute ethanol and stored for use. As controls, 1.5% Cs acetate solution and absolute ethanol were used. In the experiment, an appropriate amount of cultured test bacteria (400 μL) was evenly spread on the surface of solid agar media. Then, 6 mm diameter paper discs were placed on the agar surface, and 6 μL of different material solutions were applied to the holes in the discs. The plates were incubated at 37 °C for 12 h, and the antibacterial effects were recorded using a digital camera.

MIC experiment: *E. coli* was used as the test bacterium. No compound was added to the liquid medium for the blank control group. AAVO, AAVO-Ne, and AAVO-NeCs were added to the *E. coli* liquid medium at different concentrations in gradient steps and incubated at 37 °C on a shaking incubator for 12 h. The turbidity of the culture medium was observed, and the point at which the medium became clear was defined as the MIC.

Colony counting method experiment: In this experiment, *E. coli* was used as the test strain, and the colony counting method was employed to quantitatively analyze the antibacterial effects of AAVO, AAVO-Ne, and AAVO-NeCs. The three materials were mixed with bacterial suspension, and samples were taken at time points of 0, 5, 10, 30, 40, and 60 min. A 10 μL sample of the bacterial suspension was plated onto solid agar media and evenly spread. The plates were then incubated at 37 °C for 12 h, and the colony count was determined. The antibacterial rate was calculated using the following Formula (1):

(1) $$A=\frac{B-C}{B}\times 100\%$$

where *A* is the antibacterial efficiency, *B* is the colony count of the control group, and *C* is the colony count after treatment with antibacterial materials at different time points.

*E. coli* clearance from fruit and vegetable surfaces experiment: Fresh cucumbers were selected, cut into 0.5 × 1 × 2 cm^3^ pieces, and sterilized under ultraviolet light to remove surface microorganisms. The cucumber pieces were immersed in a solution of *E. coli* and incubated at 35 °C for 24 h. After incubation, the cucumber pieces were removed and rinsed three times with sterile water. The pieces were then treated with AAVO, AAVO-Ne, and AAVO-NeCs and incubated in the culture chamber for another 24 h. After treatment, sterile water was added to the cucumber pieces, mixed well, and 10 μL of the supernatant was taken for viable bacterial counting using the plate colony counting method.

### 3.7. In Vitro Release Experiment Investigation

A precise amount of AAVO, AAVO-Ne, and AAVO-NeCs were weighed and placed in a drying oven at 60 °C for continuous evaporation over 7 days. Every 12 h, the samples were removed, weighed, and repositioned. The loss rate of the AAVO, AAVO-Ne, and AAVO-NeCs was calculated using Formulas (2)–(4) [67]. The cumulative release rate was determined according to Formula (5).

(2) $$AAVO\;loss\;rate=\frac{m_{J}-m_{Jt}}{m_{J}}\times 100\%$$

(3) $$AAVO-Ne\;loss\;rate=\frac{m_{R}-m_{Rt}}{m_{R}}\times 16.15\%\times 100\%$$

(4) $$AAVO-NeCs\;loss\;rate=\frac{m_{y}-m_{yt}}{m_{y}}\times 16.15\%\times 60\%\times 100\%$$

(5) $$accumulated\;release\;rate=\frac{AAVO\;released\;per\;release}{The\;initial\;amount\;of\;AAVO\;added\;to\;the\;sample}$$

In the formula, *m_J_* and *m_Jt_* are the initial mass/g of AAVO and the mass/g at *t*, respectively. The *m_R_* and *m_Rt_* are the initial mass/g and the mass/g at *t* of AAVO-Ne, respectively, and 16.15% is the mass fraction of AAVO in AAVO-Ne. *m_y_* and *m_yt_* are the initial mass/g of AAVO-NeCs and the mass/g at *t*, and 60% is the mass fraction of AAVO-Ne.

### 3.8. Antioxidant Experiment Investigation

Healthy, undamaged potatoes were selected from the market, cleaned with water, air-dried, and cut into 1 × 1 × 2 cm^3^ pieces. These were randomly divided into four groups, with four pieces per group, ensuring no contact between them. Each group was coated with a solution of the same AAVO concentration using a brush, applying AAVO, AAVO-Ne, AAVO-NeCs, and an untreated control group. Once the coated surface dried naturally, the potato pieces were stored at room temperature. Starting on day 0, the weight of each group was measured every 12 h, and the weight loss rate was calculated using Formula (6) [68].

(6) $$weight\;loss\;rate=\frac{m_{k}-m_{kt}}{m_{k}}\times 100\%$$

In the formula, *m_k_* is the initial mass/g, and *m_kt_* is the mass/g at time *t*.

### 3.9. Data Statistical Analysis

All experiments were conducted in triplicates, with results expressed as the mean ± standard error. Data analysis was performed using one-way ANOVA and tests in SPSS software (IBM SPSS Statistics 25). Statistical significance between means was calculated at a 95% confidence level (*p* < 0.05).

## 4. Conclusions

In this study, it was found that AAVO-Ne prepared with 19.88% Tween-80, 3.11% anhydrous ethanol, 16.15% AAVO, and 60.86% distilled water had the best performance. AAVO-NeCs were ultrasonically combined with 1.5% Cs acetic acid solution at a ratio of 1.5: 1 at 20 °C for 40 min. After adjusting the pH value to 5, the antibacterial effect of AAVO-NeCs was the best. The results of bacteriostatic experiment showed that the three materials had inhibitory effects on the growth of four kinds of bacteria, among which AAVO-NeCs had the best bacteriostatic effect and AAVO had the worst bacteriostatic effect. The results of filter paper diffusion method showed that the three materials had the best antibacterial effect on drug-resistant Salmonella. In addition, the results of an in vitro release experiment, antioxidant experiment and bacteriostatic experiment were consistent, and the prepared AAVO-NeCs showed unique advantages. It can be seen that the AAVO-NeCs prepared in the experiment can realize the solubilization, synergism, efficient transportation, bacteriostasis, sustained release and controlled release, and antioxidant properties of VVAO.

Although we successfully prepared AAVO-related complexes with strong antibacterial activity, the molecular mechanism of how AAVO-related complexes are antibacterial, as well as their effects on bacterial cell membranes and interference with metabolic pathways, needs to be further explored. Meanwhile, AAVO-related complexes are inextricably linked to food preservation, so the effects of different concentrations and combinations of AAVO complexes on food preservation need to be further systematically evaluated.

With the increasing attention on natural medicines and green therapies, the development and application of AAVO have gained more focus. AAVO-NeCs, due to their improved bioavailability, enhanced stability, and sustained release properties, can effectively enhance their inhibitory effects against bacteria, fungi, and viruses, making them particularly advantageous in pharmaceutical formulations. This offers significant prospects for treating infectious diseases, skin care, and anti-inflammatory pain relief. The natural components of AAVO are well-compatible with the skin, and the Ne form can enhance its permeability, contributing to more effective local treatment and further boosting its clinical application value. In addition, AAVO-NeCs can also be applied in health foods, cosmetics, air fresheners, and other fields, demonstrating unique advantages in improving health and beauty effects.

## Figures and Tables

**Table 1:** Abbreviations.

Abbreviations	Detailed Interpretation
AAVO	*Artemisia argyi* volatile oil
AAVO-Ne	*Artemisia argyi* volatile oil nanoemulsion
AAVO-NeCs	chitosan composite of *Artemisia argyi* volatile oil nanoemulsion
Ne	nanoemulsion
Cs	chitosan

**Table 2:** Different SOR AAVO-Ne formed at different Km values.

Km	SOR
4:1	9:1; 8:2; 7:3; 6:4; 5:5
3:1	9:1; 8:2; 7:3; 6:4
2:1	9:1; 8:2; 7:3; 6:4; 5:5
1:1	8:2; 7:3; 6:4; 5:5
1:2	8:2; 7:3; 6:4
2:3	8:2; 7:3; 6:4

**Table 3:** Appearance and shape of AAVO-Ne.

Ratio	Appearance Shape
9:1	Clarification, clarity
8:2	Clarification, semi-clarity
7:3	Muddy
6:4	Clarification, clarity
5:5	Clarification, clarity

## Data Availability

All data are included in this manuscript.

## Supplementary Materials

The following supporting information can be downloaded at: https://www.mdpi.com/article/10.3390/molecules30030585/s1, Figure S1: Comparison chart of extraction AAVO using three methods; Figure S2: Exploration of preparation conditions for AAVO-NeCs. Figure S3: Inhibition of four bacteria by three materials. Figure S4: Exploration of minimum inhibitory concentration. Figure S5: Colony count experiment. Figure S6: Bacterial removal effect on fruit and vegetable surfaces experiment.
