# Supplementary material for: Preparation and Properties of Chitosan Complexes Consisting of Artemisia argyi Volatile Oil Nanoemulsion
Source: Molecules. 2025 Jan 27;30(3):585. doi: 10.3390/molecules30030585 (PMC11820023; doi:10.3390/molecules30030585)

Supplementary Materials

Supplementary 1:

This paper provides a comprehensive analysis of the existing literature reports, screening three commonly used experimental methods with relatively high extraction yields. The optimal extraction method is determined based on the yield of AAVO as the indicator. The specific procedures are as follows:

Steam Distillation Extraction: Accurately weigh 5 g of *Artemisia argyi* powder and place it in a 250 mL Erlenmeyer flask. Add 100 mL of distilled water, ultrasonically shake for 60 minutes, and then perform steam distillation at 110°C for 150 minutes. Transfer the distillate into a separatory funnel, add ethyl acetate for extraction. After centrifuging at 10,000 rpm for 20 minutes, collect the upper liquid and concentrate the organic phase to obtain AAVO.

Anhydrous Ethanol Extraction: Accurately weigh 5 g of *Artemisia argyi* powder and place it in a 250 mL Erlenmeyer flask. Add 100 mL of anhydrous ethanol, ultrasonically shake for 60 minutes, and then perform Soxhlet extraction at 85°C for 120 minutes. After centrifuging at 10,000 rpm for 20 minutes, collect the upper liquid and remove the anhydrous ethanol by rotary evaporation to obtain AAVO.

Petroleum Ether Extraction: Replace the solvent (100 mL anhydrous ethanol) with 100 mL petroleum ether and set the heating temperature to 60°C. The remaining procedures are identical to those in the anhydrous ethanol extraction method.

The yields of the three methods are shown in the following figure: (A represents the petroleum ether extraction method; B represents the steam distillation method; C represents the anhydrous ethanol extraction method).

Figure S1: Comparison chart of extraction of AAVO using three methods.

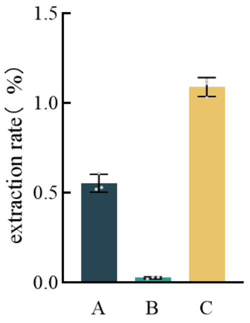

Supplementary 2:

The following figure explores the optimal preparation conditions for the *Artemisia argyi* volatile oil nanostructured chitosan complexes (AAVO-NeCs).

With the inhibition of *E coli* as the indicator, five single factors are varied for the experiment: (a) pH variation; (b) pH control (pure pH); (c) temperature variation; (d) variation of the complex ratio; (e) variation of chitosan concentration; (f) variation of complexation time.

Figure S2: Exploration of preparation conditions for AAVO-NeCs.

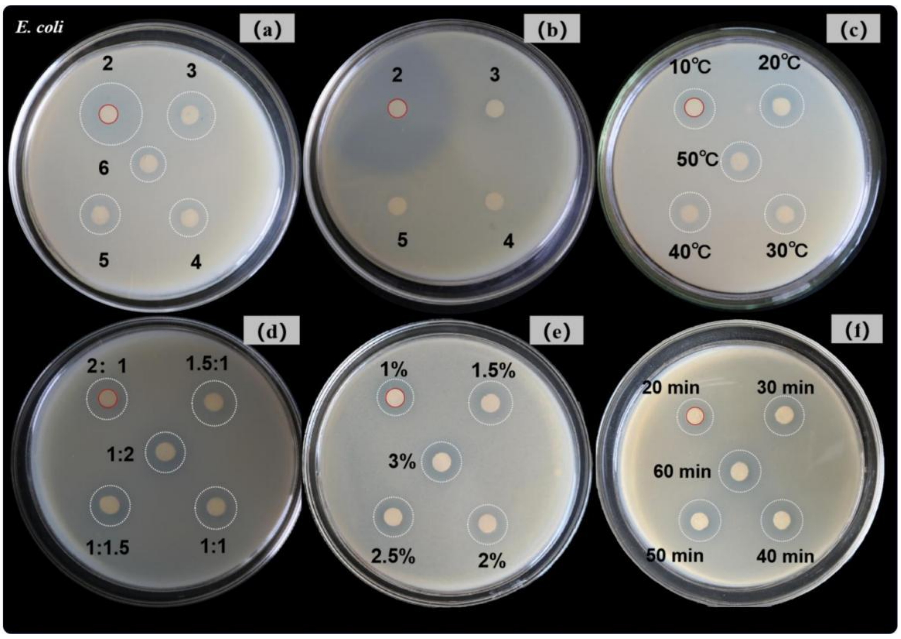

Supplementary 3:

The following figure shows the inhibition of four types of bacteria by three materials and two control materials:

In the figure, A represents AAVO; B represents AAVO-Ne; C represents AAVO-NeCs; D represents anhydrous ethanol; E represents chitosan acetate solution.

The inhibition effects of these materials on the Gram-positive bacterium *S. aureus*, the Gram-negative bacterium *E. coli*, the drug-resistant bacterium *T-Salmonella*, and the fungus *S. aureus* are investigated.

Figure S3： Inhibition of four bacteria by three materials.

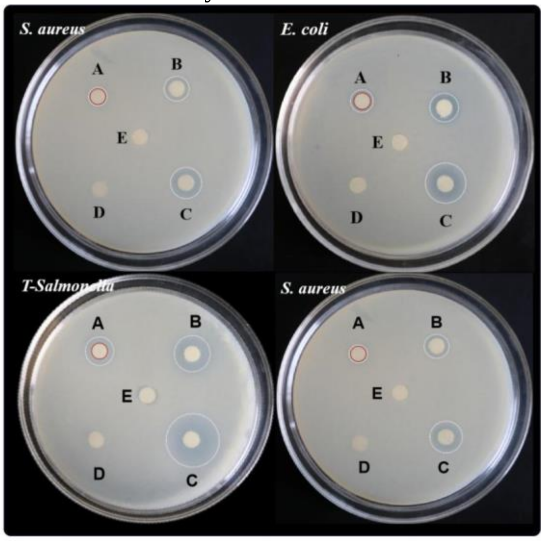

Supplementary 4:

The following figure shows the experimental picture of the minimum inhibitory concentration.

Figure S4： Exploration of minimum inhibitory concentration

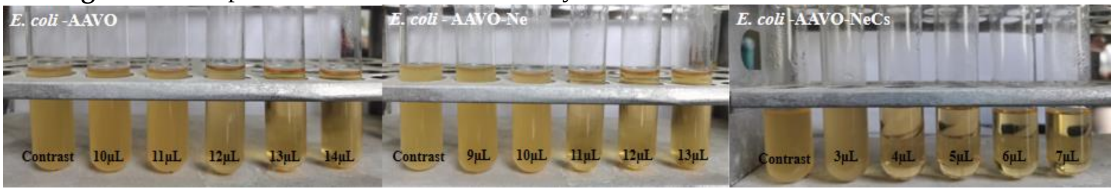

Supplementary 5:

The following figure is a picture of the colony count:

Figure S5: Colony count experiment

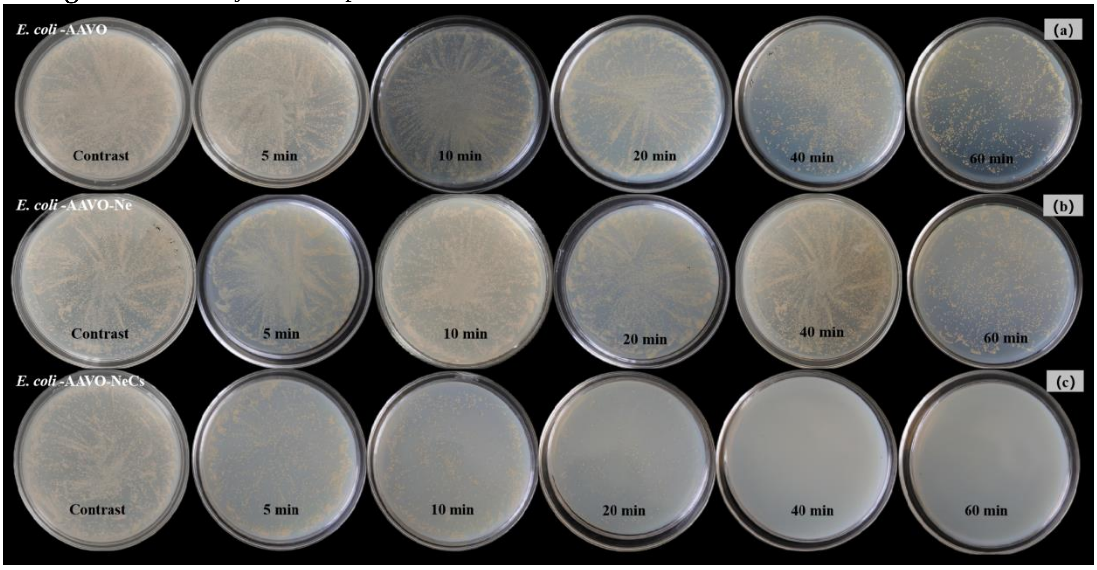

Supplementary6:

The following figure shows the removal of bacteria on the surface of fruits and vegetables:

Figure S6: Bacterial removal effect on fruit and vegetable surfaces experiment

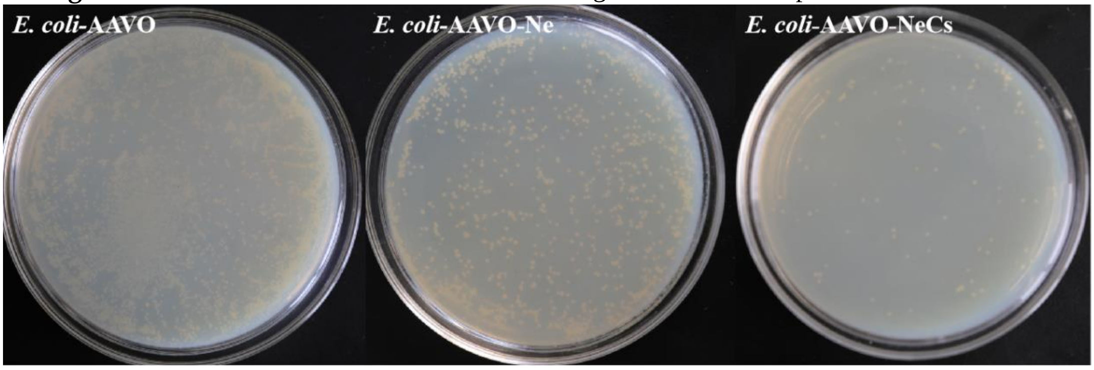

Supplement: Supplementary file 1 [file molecules-30-00585-s001.zip › molecules-3429592-supplementary.pdf]
